# Towards net zero: A technological review on the potential of space-based solar power and wireless power transmission

**DOI:** 10.1016/j.heliyon.2024.e29996

**Published:** 2024-04-24

**Authors:** Khandoker Shahjahan Alam, A.M.A. Daiyan Kaif, Sajal K. Das, Sarafat H. Abhi, S.M. Muyeen, Md. Firoj Ali, Zinat Tasneem, Md. Manirul Islam, Md. Robiul Islam, Md. Faisal R. Badal, Md. Hafiz Ahamed, Subrata K. Sarker, Prangon Das, Md. Mehedi Hasan

**Affiliations:** a Department of Mechatronics Engineering, Rajshahi University of Engineering & Technology, Bangladesh; b Department of Electrical and Electronic Engineering, Rajshahi University of Engineering & Technology, Bangladesh

**Keywords:** Space-based solar, Wireless power transmission, Solar power satellite, Sustainable energy, Solar energy

## Abstract

The global need for energy is increasing at a high rate and is expected to double or increase by 50%, according to some studies, in 30 years. As a result, it is essential to look into alternative methods of producing power. Solar photovoltaic (PV) power plants utilize the sun's clean energy, but they're not always dependable since they depend on weather patterns and requires vast amount of land. Space-based solar power (SBSP) has emerged as the potential solution to this issue. SBSP can provide 24/7 baseload carbon-free electricity with power density over 10 times greater than terrestrial alternatives while requiring far less land. Solar power is collected and converted in space to be sent back to Earth via Microwave or laser wirelessly and used as electricity. However, harnessing its full potential necessitates tackling substantial technological obstacles in wireless power transmission across extensive distances in order to efficiently send power to receivers on the ground. When it comes to achieving a net-zero goal, the SBSP is becoming more viable option. This paper presents a review of wireless power transmission systems and an overview of SBSP as a comprehensive system. To introduce the state-of-the-art information, the properties of the system and modern SBSP models along with application and spillover effects with regard to different sectors was examined. The challenges and risks are discussed to address the key barriers for successful project implementation. The technological obstacles stem from the fact that although most of the technology is already available none are actually efficient enough for deployment so with, private enterprises entering space race and more efficient system, the cost of the entire system that prevented this notion from happening is also decreasing. With incremental advances in key areas and sustained investment, SBSP integrated with other renewable could contribute significantly to cross-sector decarbonization.

## Highlights

• Reviewing the implementation and development of existing Space-based Solar Power and Wireless Power Transmission.
• The general Space-based Solar Power framework and models.
• The possibility of Space-based Solar Power as Sustainable energy.
• Challenge and outcome of integrating Wireless Power Transmission and Space-based Solar Power with traditional grid.

## Graphical abstract

**Figure:**
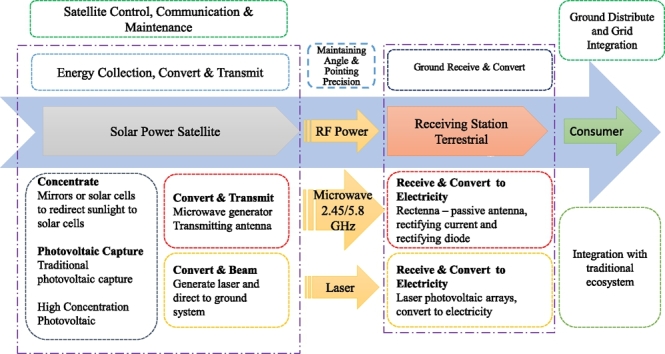

## 1 Introduction

Worldwide energy consumption is expected to double or increase by 50%, according to some studies, in 30 years [1]. The impact of climate change on land and resources is equally significant [2]. Now is the time for sustainable, inexpensive, scalable, and secure clean energy. Due to the increased demand for energy, the rate of pollution growth is accelerating at a rate never before seen. This rise is due to the use of coal, natural gas, and oil to produce energy [3]. Traditional energy production methods emit hazardous acid gases, mercury, non-mercury toxics, and organic air toxics. 50–60% of mercury, 75% of acid gases, and 20–50% of hazardous metals are produced by power plants in the US. According to a study, power plants release 10B tons of CO_2_ annually [4]. We must prioritize the generation of renewable energy and a sustainable society [5].

The sun is the primary energy source, in this solar system. 70% of solar energy that reaches the earth's surface is lost due to the day-night cycle and the inability to efficiently utilize solar energy [6]. The efficiency of the most modern solar cells is just over 40%, whereas the efficiency of the most common solar cells ranges between 22% and 27% [5]. To address these issues, scientists have investigated space-based solar power (SBSP) for decades. This concept entails launching solar power satellites (SPS) into orbit in order to collect and transmit solar energy [6].

In 1968, scientists initially proposed this “space solar-power system” (SSPS) [7]. Due to a lack of adequate technology and the expensive cost of space flight, this issue has been the subject of numerous unsuccessful projects since 1970. Reusable rockets and technological advancements have prompted a rethinking of this subject [8]. The most challenging aspect of the project is the technological aspect. The satellite may exceed one kilometer in size [9]. After construction on Earth, this enormous system is too hefty to transport into space. Innovative, lightweight, cost-effective, and efficient solar cells are required [10]. People aren't always open to new and uncertain technologies, either [11]. These variables make this endeavor fascinating and challenging. This study examines the technological obstacles and prospects of space-based solar power, as well as SBSP's current microwave power transmission research.

**Figure 1:**
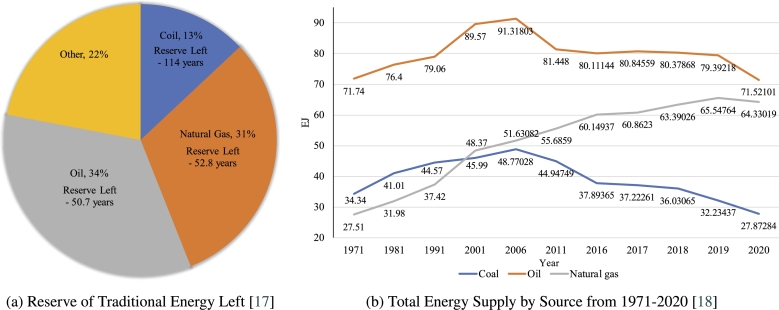
Traditional Energy Reserve & Supply Scenarios [17], [18].

**Figure 2:**
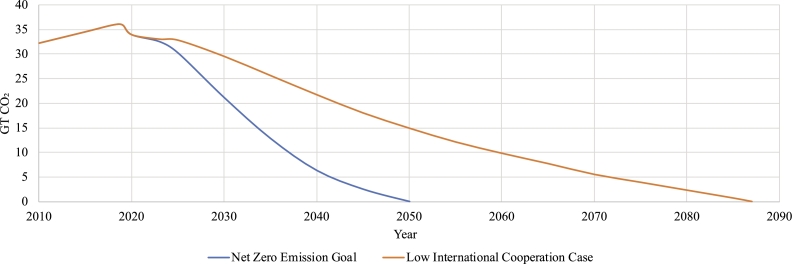
CO_2_ emissions scenarios over time for net zero goal and low international cooperation case [16].

In the Paris Agreement, Net Zero 2050 was proposed. To restrict global warming to 1.5 °C, emissions must decrease by 45% by 2030 and reach zero by 2050, according to the report [12]. These goals have been prioritized by several nations. Yet, this objective is not even close to being achieved. To achieve this, renewable energy and sustainable technology must become the norm. Since the majority of nonrenewable energy sources, with the exception of nuclear power, have limited supplies, we must reduce our reliance on them over the coming decades. We are running out of nonrenewable sources of energy. Fig. 1a depicts the estimated energy supply left, whereas the Fig. 1b illustrates our reliance on coal, gas, and oil and the rate at which they are depleting. Nonrenewable fuels also hinder net-zero emission goals. The International Energy Agency (IEA) provides guidance to assist nations in accomplishing the goal. This guideline establishes emission targets for various years and common goals to achieve them [13]. Although governments are committed to achieving net-zero emissions by 2050, the current regulatory framework and industrial landscape cannot be adjusted quickly [14], [15]. Fig. 2 highlights the milestones for achieving net-zero emissions by 2050 as well as how low international collaboration (with current policies) pushes it to 2087 [16].

**Figure 3:**
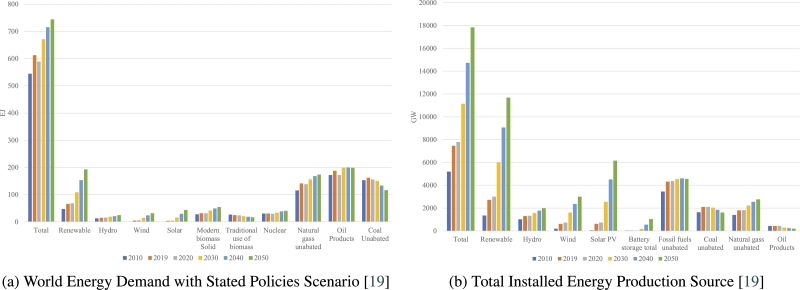
World Energy Demand & Capacity Scenarios [19].

Fig. 3a depicts the global energy consumption under existing policies. As energy consumption slowly increases under the current policies, so does interest in renewable sources, but a deeper look at the other sources reveals the real issue. The demand for renewable energy is growing rapidly, whereas the consumption of nonrenewable energy stays stable. Installed capacity is the maximum electricity-generating capability, whereas energy demand is comprised of multiple components [26]. Fig. 3b demonstrates that renewable production capacity will expand fast in the near future, whereas nonrenewable sources are not decreasing at a comparable rate. Conventional renewable sources are underutilized in many nations because of their insufficient efficiency, electricity-generating capacity, and government assistance [27]. Renewable sources are yet to be superior in terms of production capabilities and overall efficiency, despite having unlimited resources [28]. This also demonstrates that growing renewables alone may not be sufficient to reduce the use of fossil fuels or assist countries in transitioning to a greener economy [29]. Several SBSP review papers have been published. Our paper is compared to these review studies in Table 1. In the later section, we talk about the importance of this paper and what we hope to demonstrate.

**Table 1:** Table of Discussed Topics in Available Review Papers.

Author(year)	Net zero & SBSP	SBSP Evolution	Framework	Wireless Power Transmission	SBSP applications	Challenges & Risks
Gerardo Antonio Urdaneta et al (2022) [20]	-	-	✓	✓	-	-
Chad M. Baum et al (2022) [21]	-	✓	✓	-	✓	✓
Tao Zhang et al (2021) [22]	-	✓	✓	-	-	✓
Paul Jaffe et al (2019) [23]	-	✓	✓	✓	✓	-
Muhammad Badar Hayat et al (2019) [24]	-	✓	-	-	-	✓
W. N. Johnson et al (2009) [25]	-	-	✓	✓	✓	✓
Current Paper	✓	✓	✓	✓	✓	✓

Space solar can solve this renewable energy supply conundrum and assist in reaching net zero by 2050. Solar energy obtained from space can provide safe, sustainable, environmental friendly, and economical electricity wherever on Earth. Humanity can transition away from fossil fuels with the aid of space solar power. This will significantly reduce our reliance on nonrenewable resources. Clean baseload electricity that is available regardless of location, time, or weather is the objective of SBSP [30]. This study examines space-based solar power technology, its obstacles, and its potential benefits. It investigates the structure, components, significance, and necessity of SBSP for a sustainable future. The origins, existing actions, and many conceptions of SBSP are discussed. This research examines wireless power transmission methods, such as SBSP's microwave technology. This paper also addresses SBSP's applications in the energy industry and other industries, as well as its challenges and risks, and suggests viable solutions.

## 2 Space-based solar power: evolution & crests

**Figure 4:**
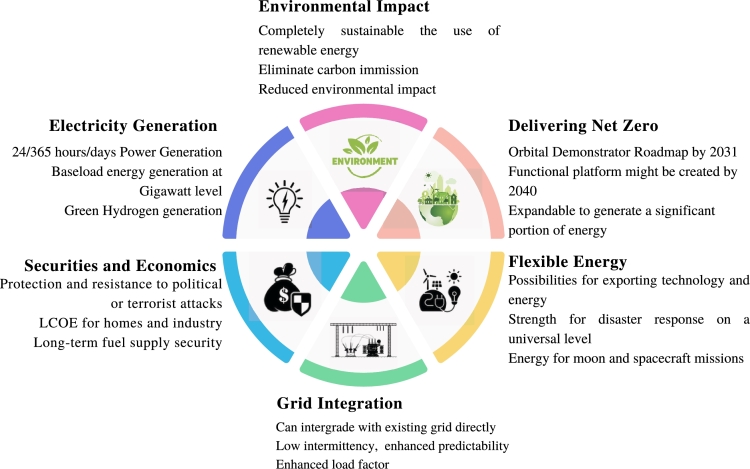
Major features of Space-based Solar Power.

Long thought of as an appealing yet pricey source of renewable energy, advocates of space-based solar power claim that this dream has now become economically and technically feasible over the past decade. Several factors contribute to the growing worldwide interest in space-based solar power. There are several specific qualities that are essential given the pressing demand for fresh sources as defined by Sustainable Development Goal 7 (SDG7). SDG7 aims for “affordable, dependable, modern and sustainable energy for all” by 2030 [31]. Assuring cheap, dependable, and widespread energy security, significantly increasing the amount of renewable energy production, energy mix, and doubling the pace of progress in renewable energy efficiency are the three main objectives of the targeted goals on energy, or SDG7 [32]. The SDG7's many aims contribute to the accomplishment of other SDG goals, and more researchers have lately turned their attention to this [33], [34]. Some of these include the need for new energy sources to be risk-free and technically possible [35], [36]. A scene of dependability and safety ought to exist [37], [38]. The system should be designed with human health in mind [39]. The system must be effective, powerful, and have a large capacity [40], [41]. The impact on the environment and climate change ought to be minimal [42], [43]. The affordability of electricity, operating and maintenance costs, as well as investment expenditures, should take precedence in the economic situation [44], [41]. Impact on human health should be kept in mind [39]. SBSP is carbon-neutral and removes key emissions from power generation. SBSP seems to be the only source of energy that offers baseload energy in a secure, dependable, and waste-free system that is green, sustainable, and efficient. Before going into more detail, we should take a look at its features, which are presented in Fig. 4.

**Figure 5:**
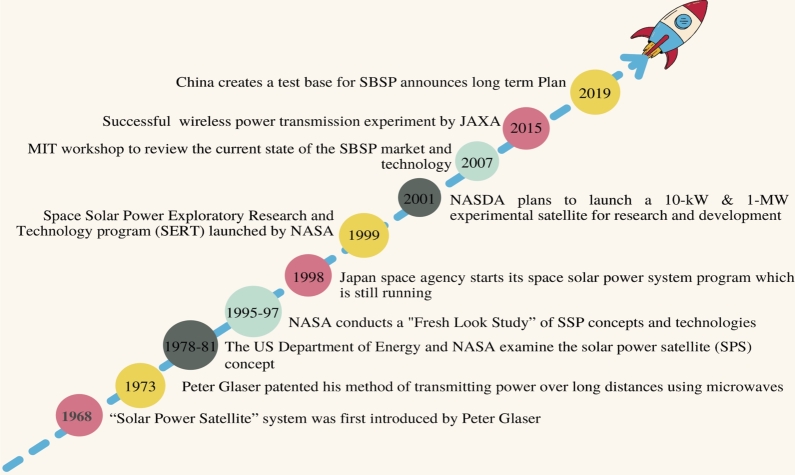
Timeline of Space-based Solar Power Development [45].

The concept of utilizing space to generate electricity originated in Isaac Asimov's short story “Reason,” in which a space station uses microwaves to transmit solar energy to multiple planets. After that, beginning in 1968, the concept evolved continuously. Fig. 5 depicts the most significant developments in space-based solar power. Since 1979, numerous concepts for solar energy collection systems, such as Integrated Symmetrical Concentrator (ISC), Omega, ALPHA, CASSIOPeiA, etc, have been proposed. In Table 2, various SBSP Models are described with their characteristics.

**Table 2:** Table of Space-based Solar Power Proposed Models.

Year	Concept	Organization	Power	Orbit	Mass (MT)	Modularity	Frequency (GHz)	Rotary Joints
1979	Reference model [46]	NASA	5 GW	GEO	30000 - 50000	No	2.45	Yes
1993	Sps 2000 [47]	ISAS	10 GW	LEO		Yes	2.45	Yes
1995	Sun tower [48]	NASA	100-300 MW	MEO	2000 – 7000	Yes	5.8	Yes
1997	SOLAR DISC [49]	NASA	1–10 GW	GEO	8000 - 70000	No	5.8	
1998	ISC [50]	NASA	1.2 GW	GEO	22463	Yes	2.45	No
1999	Sail Tower [51]	ESA	450 MW	GEO	2100	Yes	2.45	Yes
2001	Tethered [52]	USEF/METI	750 MW	GEO	3800	Yes	5.8	No
2012	ALPHA [53]	ARTEMIS	2 GW	GEO	25260	Yes	2.45	No
2015	OMEGA [54]	Xidian Univ.	2 GW	GEO	22953	Yes	5.8	Yes
2015	MR-SPS [55]	CAST	2 GW	GEO	10000	Yes	5.8	Yes
2017	SPS-ALPHA Mk-II [56]	MANKINS Space Technology	1-2 GW	GEO	9200	Yes	2.45/5.8	Yes
2017	CASSIOPeiA [57]	IECL	2 GW	GEO	2000	Yes	2.45	Yes

Space launches have become cheaper recently, and this trend is expected to continue [66]. The private sector has added a new dimension to the economy [67]. Concepts like SPS Alpha and CASSIOPeiA are flexible, and designed for large-scale commercial production [68]. High-concentration photovoltaic (HCPV) solar panels, wireless transmission, and space robots are some of the ways it is using technology to overcome technical problems [69], [70]. Governments could see the strategic benefit and international influence in the ability to generate a supply of plentiful, inexpensive energy that could be beamed anywhere on the planet [68]. Many nations are developing space-based solar power systems. US, UK, China, and Japan have major national policy-driven projects [71]. All sides have a genuine and strong desire for cross-border collaboration. Private and government organizations are working on various project components. Table 3 summarizes SBSP development and government activities in this sector.

**Table 3:** List of Recent International Activities.

Country	Activity
USA	AFRL and Northrop Grumman Demos space-based solar power System [58]. NRL successfully beams 1.6 KW of power over 1 km wirelessly [59]. Caltech receives 100M dollar project for Space Based Solar [60].

JAXA	Research on space-based solar power has been funded by Japan, with a primary focus on WPT. JAXA successfully demonstrated wireless power transmission of 1.5 kW over 50 m [61].

China	CAST has a roadmap in place for generating 2GW of power from SBSP by 2050 in four phases. Two years ahead of schedule, China plans to start the first stage of an ambitious solar power plant development in 2028 and is planning the construction of a megawatt-scale power production facility by 2030 [62].

UK	Over fifty British companies and educational institution have joined the UK Space Energy Initiative, which was created to study ideas for developing a space-based solar plant to assist the UK reach net zero by 2050 [62]. According to a recent analysis by Frazer-Nash Consulting, the UK government is considering a £17 billion SBSP development project [63].

Europe	Over the last two decades, the ESA (European Space Agency) has regularly undertaken research into space-based solar power [64]. Recently, ESA released a request for research proposals on space-based solar power systems [64]. ESA has also conducted a workshop on SBSP [65].

## 3 SBSP: framework and architecture

The three basic components of space-based solar energy are:

1. Solar energy collection from space using photovoltaic (PV) cells or mirrors [72], [73].
2. Utilizing a laser or microwave for wireless power transmission to the Earth [74].
3. Earth-based energy capture station like a microwave antenna (rectenna) [75].

The system as a whole faces a lot of different challenges. The gravitational force exerted by the Earth diminishes as the distance from it increases. However, celestial structures in space are still influenced by gravitational disturbances originating from celestial bodies such as the Moon and the Sun. However, space debris and solar radiation would be one of the main causes for concern [76]. Due to the size of the system, it is essential to have a lightweight, large-scale framework that can support all of the system's components and can be constructed by robots without compromising the structure's rigidity [77], [78]. When launching a structure into space, orbital location is the primary consideration.

The altitude of a Lower Earth Orbit (LEO) is approximately 2000 km or below [79]. To ensure uninterrupted energy conversion throughout the day for a specific point on Earth a cluster of stations, consisting of 3-13 modules, must be deployed or multiple countries would need to collaborate on a project to reduce the module numbers but that would interrupt 24/7 supply. The gravitational force is stronger in Low Earth Orbit, which necessitates the use of additional fuel for station maintenance. The orbital period of satellites in Medium Earth Orbit (MEO) ranges from 6 to 12 hours. This satellite would maintain a more prolonged presence over a particular region of Earth [80].

The altitude of the Geostationary Earth Orbit (GEO) from Earth is roughly 36,000 km. Once a satellite or SBSP module is placed into this orbit, it will orbit the Earth at the same speed as the Earth's rotation [79]. As a result, it will always remain in the same position where it was initially launched, even though testing will commence at a lower Earth orbit. Due to its stationary position, the majority of concepts are typically centered on the Geostationary orbit (GEO), but testing must begin at much lower orbits. This will help maintain a nearly constant base load throughout the year by using only just one module to provide power to a single location on Earth. The cost of launching into GEO is unquestionably more compared to that of LEO and MEO [80]. The efficiency of a double-sided PV cell solar plant surpasses that of the MEO by implementing precise in-orbit module movement with the application of minimal thrust which is required for orbit stabilization and is quite minimal compared to the other two orbits [79], [81]. Therefore, it can be regarded as an optimal orbit for launching SBSP.

**Figure 6:**
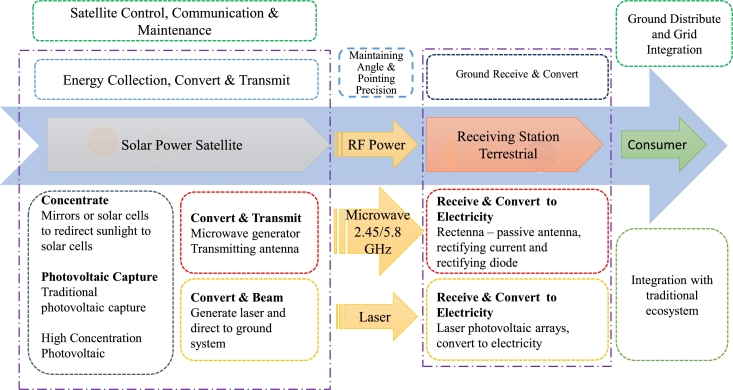
System Structure Diagram.

**Figure 7:**
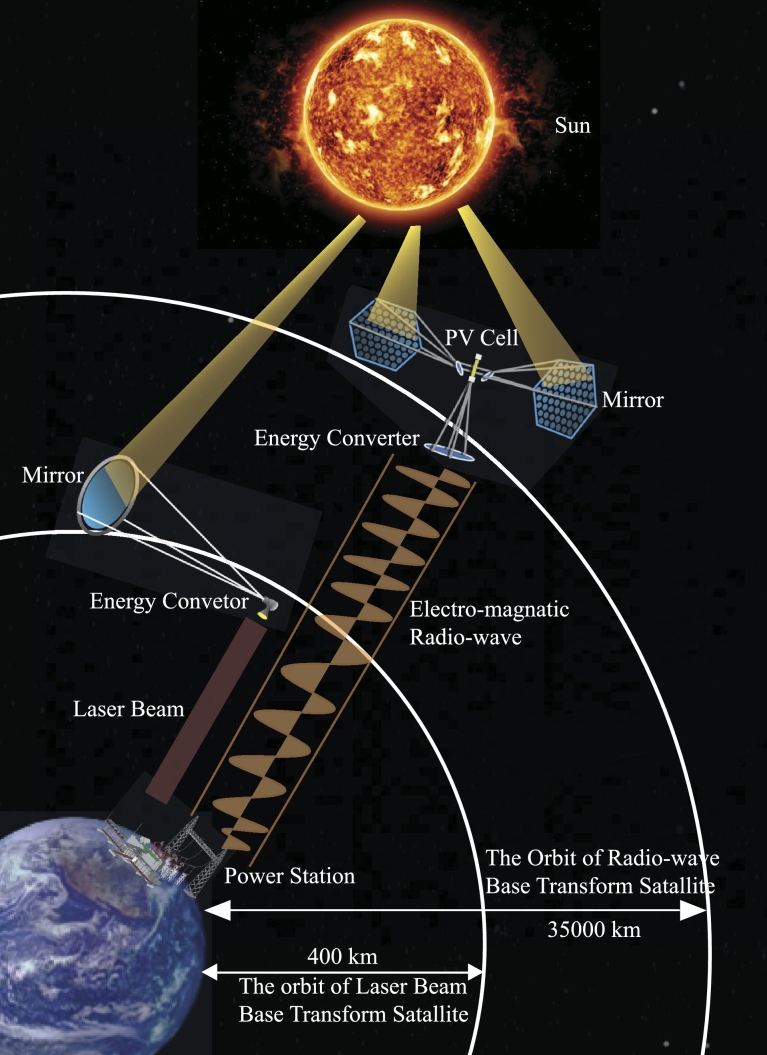
Overall System Structure.

For wireless transmission microwave-based system is applicable in all orbits, whereas the laser-based system is restricted to lower orbits. The disadvantages of laser-based technologies extend beyond safety, environmental, and legal concerns. For these and other factors, microwave-based transmission is the primary focus of research. For microwave transmission, photovoltaic (PV) and solar dynamic (SD) energy conversion methods are being researched, with PV technology taking precedence [82]. In PV technology, the solar rays are captured in the form of energy like traditional solar cells and then converted to energy [83]. PV uses semiconductor materials like silicon to directly convert sunlight to electricity. Their simplicity, low maintenance needs, and adaptability to diverse environments make them a reliable choice for space applications. However, PV systems face challenges such as limited efficiency, considerable weight, and sensitivity to temperature fluctuations. Solar dynamic systems use mirrors to concentrate the light onto a working fluid, subsequently generating electricity through a heat engine. Using mirrors might have been a viable solution for reducing the overall mass of the system, but this has its own complications [84]. Nevertheless, their intricate design, reliance on moving parts, and sensitivity to precision in pointing pose challenges in terms of complexity and maintenance. Due to the proposed frequency, wireless power transmission using microwaves is safe for humans [85], [86]. The beam locks onto the ground to transmit to the rectenna. The ground-based rectenna converts microwaves to electricity that can be modified and delivered to the grid with the appropriate properties. The entire system is divisible into numerous subsystems. The subsystems for microwave-based SBSP systems are discussed in Fig. 6. Fig. 7 illustrates the whole system view of the diagram (presented in Fig. 6) for a better perspective. The following are the subsystems for microwave-based SBSP systems:

### 3.1 Energy collection & conversion

Collecting solar energy would be the primary function of the system's first stage. Solar cells or mirrors would be employed on that scale (possibly kilometers). The collected photons must be directed toward the conversion mechanism. This is one of the most challenging aspects of the system, as current solar cell technology falls short of expectations. Before solar energy can be transmitted, it must be converted from electrical energy microwave or leaser. This will also include the necessary power conditioning prior to transmission in order to increase efficiency. New developments in power electronics have streamlined the conversion procedure, although more efficiency still needs investigation. [87]. HCPV will aid in reducing system mass and increasing conversion efficiency, heat deception and decreasing system mass [88].

### 3.2 Energy transmission

This subsystem uses microwave power beams to transmit large amounts of power. This involves converting electrical energy into non-ionizing radio frequency and transmitting it from the satellite to the receiving station [89]. The production of big, effective power transmission antennas must be a key focus. These technologies are inefficient at small scales and are impossible to verify unless they are implemented on a large scale. Although some testing has been conducted, more intensive research is necessary to improve the system's efficiency and accuracy [90]. The production of a coherent beam on a flexible framework will be difficult. This is one of the most serious technological obstacles to the development of space-based solar power. Frequency bands must be assigned and agreed upon at the international level to prevent interference with existing systems.

### 3.3 Satellite control, communication & maintenance

Satellite maintenance and communication account for a large amount of activity. This comprises thermal management, station maintenance, communication, and control systems for satellites. To enhance performance and dependability, power electronics and other components must be adequately cooled [91]. Further testing is needed for thermal load and heat dissipation in space. The need for cooling systems in space is debatable. Space thermal load and heat dispersion need more testing. In this instance, contemporary satellite technology may aid in developing a deeper understanding. To maintain the requisite orbit, the spacecraft would require thrusters [92]. Massive structures face scaling and technology refinement issues. Huge constructions require better technology. Maintaining the station while lowering structural bulk requires electric thrusters and mechanical damping.

The control of satellites requires the integration of sensors, control logic, and processing. The control system is anticipated to be considerably more advanced than existing satellites [93]. It is anticipated that additional sensors will be needed to help control systems and respond to unexpected threats. Developing resource management methods for regulating the antenna beam while keeping system security in mind will be necessary [94]. Constant communication is required to control and monitor systems from Earth. Given how far contemporary communication technologies have progressed, communications systems will be among the least difficult aspects of the system as a whole [95].

### 3.4 Sun-Earth pointing

#### 3.4.1 Maintaining angle

Throughout its orbit, an SBSP in GEO maintains the same angle with respect to the Earth. In GEO orbit, the angle between the sun-facing solar collector and the Earth-facing microwave transmitter always changes. Consequently, if the solar collector constantly aims toward the sun, the microwave beam must always be aimed at the right place on Earth. If the microwave beam is set to aim toward the Earth, then the collector must constantly face the sun [96]. To overcome this difficulty, some solutions use mechanical steering, while others employ solid-state electrical beam steering.

#### 3.4.2 Pointing precision

For designs utilizing mirrors as collectors, the mirrors must be aligned with extreme precision in order to maintain high solar intensity on the PV components. The mirrors must keep homogeneity high enough to prevent localized hotspots from forming on the PV components [97].

### 3.5 Ground receive & convert

Ground systems will be easier to construct and maintain in comparison to space systems. The rectenna, which turns the transmitted beam into electrical energy, is the principal component of the ground receiving system. This is difficult due to our lack of skill and knowledge of significant rectenna technology and our inability to conduct experiments on Earth's orbit. To decrease the size of the space structure, a huge antenna area is necessary on Earth. A smaller rectenna might be utilized, but it would collect only a portion of the energy being sent. The size of the satellite aerial and Earth's rectenna are connected due to diffraction physics. Tests have demonstrated that the concept is theoretically feasible [98], [99], [100], [101], [102]. Transforming electricity into a form suitable for feeding the grid requires, grid operability techniques such as power conditioning and reactive power control. SBSP and PV stations use similar power conditioning, inverting, and reactive power management techniques.

### 3.6 Ground distribute & grid integration

Using transformers and transmission lines to collect electricity from the site and deliver it to the power grid is the easiest step preceding conversion. The infrastructure to connect such technology to the grid already exists. A control station will manage the satellite, the transmission of power, the conversion of power, the distribution of power, and all data flow through and be monitored.

### 3.7 Laser based

The concept for laser-based systems is nearly identical. In lower orbit, the satellite will pass through multiple countries. Either the project will be a multinational endeavor, or energy will not be harvested around the clock. Solar energy will be converted to laser beams by solid-state laser systems. This laser beam is directed towards the ground station, transforming its energy into electrical energy [103], [104].

### 3.8 Other factors affecting the system design

#### 3.8.1 Cost & levelized cost of electricity (LCOE)

There are numerous variables that affect the total cost. There are also construction and maintenance expenses. The costs of operation and maintenance, as well as the system's expected lifespan, must be considered. Overall cost is required to calculate LCOE, which is another critical factor. This is a fundamental indicator for comparing the total lifetime cost of electricity production. It is the ratio of a power plant's total lifetime cost to its total energy output over the lifetime period [105], [106], [107].

According to [108] the estimated cost of CASSIOPeiA will be £16.3 billion with an LCOE of 50£/MWh and [109] states the estimated cost of SPS-ALPHA to be 11.4 billion dollars with an LCOE of 5.50$ per watt. But the study [108] was conducted on 2021 and [109] on 2017 and for SBSP a large portion of the cost is transportation cost which is decreasing everyday as more private companies join the space race. So, unless a project is actually started the cost cannot be properly quoted and the LCOE cannot be calculated accurately. Another fact is LCOE for different resources varies in different country based on their geography and weather. Like for large scale solar the LCOE in UK is around 33 pound/MWh but for US it varies from 24 - 96 dollar/MWh varying from state to state. For offshore wind this LCOE is in UK 33 pound/MWh and in 72 - 140 dollar/MWh US [108], [110].

#### 3.8.2 Mission flexibility

The design of the satellite is based on the target orbit, such as GEO, to get the most power and use that power over the whole orbit. Others are more adaptable and might employ multiple orbits, such as LEO and MEO, while still achieving high utilization and providing inexpensive energy. This feature may enable mission flexibility and the chance for smaller-scale development satellites to supply power for various uses (e.g., research organizations in polar regions where the cost of fuel is very high, disaster relief, and military deployments) [77].

#### 3.8.3 Materials & technology innovation

The materials and technology utilized in SBSP present one of the biggest obstacle. Evaluating these sectors reveals that while the necessary technologies are currently available, but performance still needs to be addressed. Space stations and other satellites currently employ solar panels, and while lightweight solar cells based on gallium arsenide (GaAs) are commercially available, their efficiency is about 30–35% which is still insufficient for these kinds of applications [111].

In regards to wireless transmission, Nikola Tesla conducted the first experiments at the close of the 19th century, but he was unsuccessful due to wireless power diffusion, which is dependent on the antenna's frequency and size [112]. W. Brown developed the first rectenna array concept at the Raytheon Company in 1963. Brown was successful in the WPT field tests, but the system's size and expense prevented any feasible practical uses [113].

In terms of rectenna the size and cost has always been the problem but recent experiments suggest that this is feasible. On March 3, a group of researchers at Caltech demonstrated wireless power transfer using an array of lightweight, flexible microwave power transmitters powered by specially designed electronic chips that were constructed utilizing affordable silicon technologies. To beam energy to targeted areas, it made use of an array of transmitters [114]. Experiments like these are becoming more common as electronics technology becomes more efficient.

## 4 SBSP conceptual designs: a technical review

Some concept satellite designs are being worked on by various organizations. Some of the recent concepts are discussed below.

### 4.1 Sandwich module

**Figure 8:**
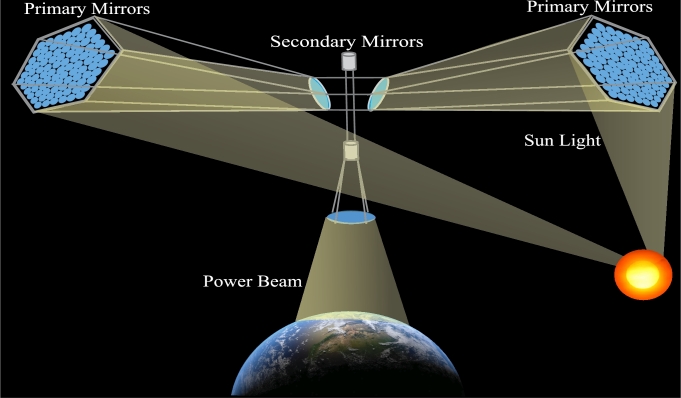
Sandwich Model Concept.

The “sandwich module,” which was first studied in connection to NASA/DOE research of the late 1970s, is the main component of the several modular SBSP layouts. The sandwich module performs tasks that can be broken down into three layers: accumulating solar energy and converting it to dc electricity; producing a radio wave signal with the proper magnitude and frequency for transmission, and creating a spaceborne transmission array aperture with sufficient beam coupling to transfer useful energy to the ground [115], [9]. A simple, basic illustration of a sandwich module is shown in Fig. 8.

### 4.2 Constant aperture solid-state integrated orbital phased array (CASSIOPeiA)

**Figure 9:**
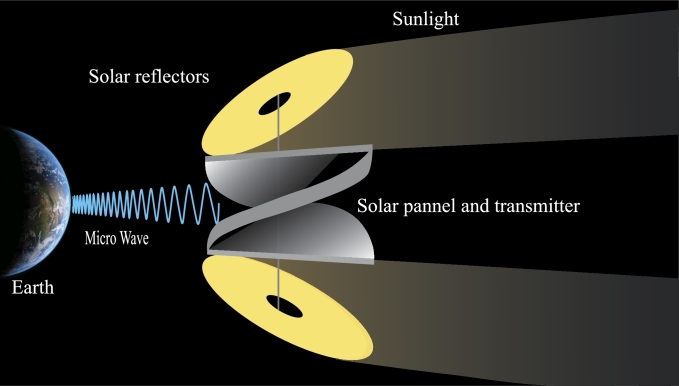
CASSIOPeiA Concept.

High Concentration Solar Photovoltaic (HCPV) panels mounted on a helical structure called CASSIOPeiA, is oriented north-south to capture solar ray reflected by mirrors at both sides of the structure. A collection of microwave emitting antennas constituting an orientable phased array are orthogonal to the HCPV panels. This enables 360° steering of the microwave beam. Because of this, the mirrors may continue to face the Sun throughout its orbit and continue to provide electricity to Earth. The system has no moving components and is entirely solid state. By adjusting the solar pressure on the mirrors using electro-chromic material, orbit stability is accomplished. Individual modules each have a combination of PV and RF dipoles, making it a modular dispersed design. Its construction from a sizeable number of only five common module types enables deployment along with robotic construction. The dispersed design was created to ease the challenges that arise from heat management and power distribution. Since there are no more single points of failure, gentle degradation is also possible. The satellite will be maintenance-free, with a provision for elegant aging during its lifespan, according to the designer. Fig. 9 depicts the CASSIOPeiA design concept.

According to Cash, the estimated mass of a 2 GW system is 2,000 tonnes, with a 1.6 km wide antenna that will beam to a 5 km rectenna. CASSIOPeiA is projected to achieve a high specific power rating, which is enabled by the arrangement's use of HCPV, which reduces the amount of PV that is needed, and the creative layout, which maximizes the usage of all the modules [116].

### 4.3 Solar power satellite via arbitrary phased array (SPS-ALPHA)

**Figure 10:**
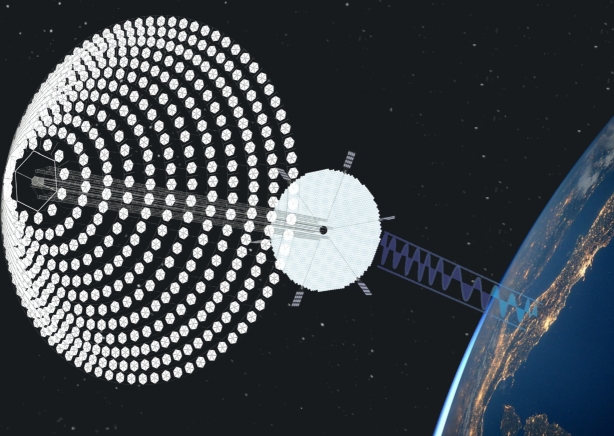
Solar Power Satellite by means of Arbitrarily Large Phased Array Concept.

SPS-ALPHA is a concentrator design that utilizes a gravity gradient-stabilized design to isolate the mirror mass from the sandwich panel expand by several kilometers. The sandwich panel functions similarly to the one on CASSIOPeiA, while being thinner. Permanently aimed towards Earth for electricity transmission. To reflect light onto the photovoltaics in the sandwich panel, this design employs heliostats that are motorized and individually adjustable in reaction to the Sun's changing position relative to the Earth. By adjusting the location of the satellite operator, the quantity of light striking the panels may be regulated. Heliostats are important for controlling thermal loads. 10 kinds of standard modules might facilitate deployment, robotic assembly, and maintenance. By eliminating single points of failure, this design enables for gentle deterioration. The design provides 2 GW of microwave power with an antenna with a diameter of 1.7 km combined with a 6 km broad rectenna. The mass is estimated to be 8,000 tonnes. To provide consistent power delivery, the satellite is intended to orbit from GEO. It is expected that the design will have a 100-year lifespan, with component replacements possible due to its flexibility [56], [96]. Fig. 10 illustrates an SPS ALPHA design concept.

### 4.4 Multi-rotary solar power satellite (MR-SPS)

**Figure 11:**
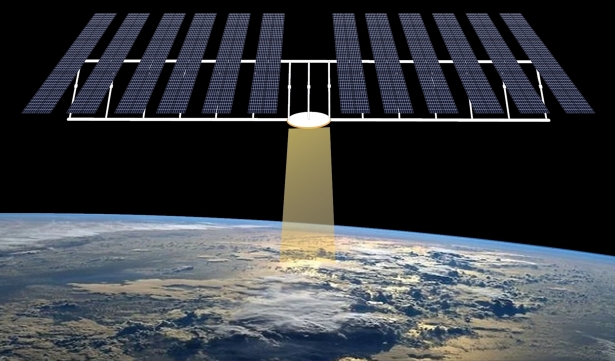
MULTI-ROTARY SOLAR POWER SATELLITE (MR-SPS) Concept.

MR-SPS is a Sun-oriented design without a concentrator. Each of the satellite's enormous solar panels is supported by a rotary joint (thus “multi-rotary”). An antenna is linked with the structure. The rotating joints let the solar panels spin in one axis independently of the framework. The power produced by the panels is sent to an antenna through the spinning joints. This technology is said to eliminate the problem of creating a precision solar concentrator setup and controlling the temperature. Moreover, the engineers recognize that the extraordinarily strong rotating joint and the vast power distribution system pose substantial technological challenges. There seems to have been much attention put into overcoming these difficulties. The current plan is for a 1 GW, 11.8 kilometer-wide, 10,000 tonnes structure in GEO. The antenna has a 1 km diameter along with a 5 km wide rectenna. The expected lifespan is thirty years [117], [91]. Fig. 11 depicts an MR-SPS design concept.

## 5 Wireless power transmission

**Figure 12:**
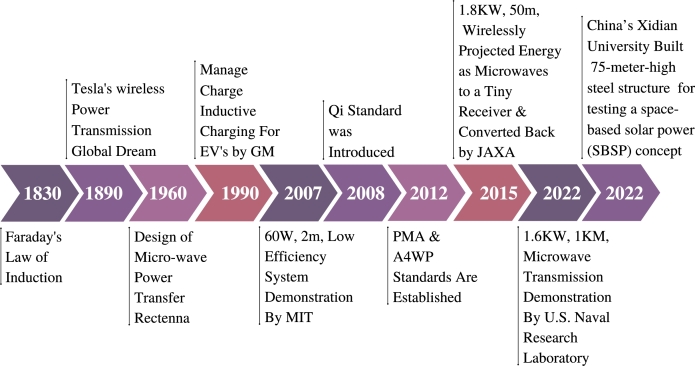
Timeline of Wireless Power Transmission [121].

Wireless power transfer (WPT) is a method for sending electricity without cables. This unique approach transfers electrical power across walls, water, air, or space between the energy source and the receiving equipment. This method uses antennas to wirelessly send and receive electricity. Solar power satellites, EVs, wireless grids, and other technologies need wireless charging. WPT is an old concept that has recently acquired popularity. Before discussing microwave power transmission, in Table 4 we explore wireless power transmission classification. WPT has been around for 200 years illustrated in Fig. 12. It works similarly to microwave ovens, laser scanners, and X-ray machines.

**Table 4:** Table of Overall Classification Wireless Power Transmission [118], [119], [120].

Categories	Transferring Method	Transmitter	Receiver	Frequency Range	Range	Efficiency	Others
Non-Radiative/ Coupling	Magnetic field/ Induction Coupling (Electro Magnetic Induction)	Copper coil	Copper coil	87 kHz - 205 kHz	Short	Low	Charging pad design dependent Less load-location flexibility
	Electric field/Resonance coupling (Displacement Current)	Primary and secondary copper coil	Copper coil	6.78 MHz for power transfer & 2.4 GHz for control signals	Medium	High	Multi-device charging Applicable to portable devices

Radiative	Microwave (or) radio wave power transmission (Electromagnetic Wave)	Transmitting Antenna with a wave guide	Rectenna	300 MHz - 300 GHz	Long	High	Omnidirectional RF spectrum for mobile devices Limits load placement flexibility
	Transmitting optical antenna	Receiving optical antenna	Millimeter to Micrometer wavelength	Long	Medium	Transmitted across extensive distance	Require receiver line of sight

### 5.1 Wireless power transmission for SBSP

Wireless power transfer will make up a sizable portion of the system. A laser or microwave will be used to send the stored energy to the earth's surface. These are described in the sections that follow.

#### 5.1.1 Laser

**Figure 13:**
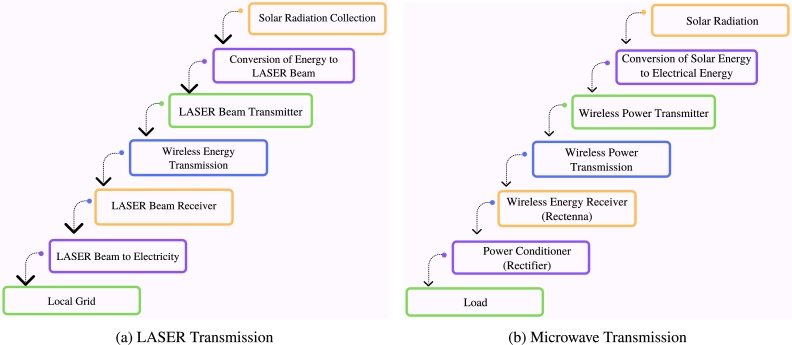
Block Diagram of Wireless Power Transmission.

Fig. 13a shows the basic schematics of a laser-based SBSP system. Utilizing solid-state laser systems, the energy will be transformed into a laser. Laser power transmission functions by employing highly effective photovoltaic cells at the receiving end to capture the laser beam emitted by the transmitter, turn it into electric energy, and energize the laser light. The laser source transmits energy through a highly effective lens. This lens focuses the laser beam precisely onto the designated spot of the receiver. The base station converts the beam energy into power. The problem is solar laser production and energy conversion. Lasers in space can blind and weaponize nations. Efficiency is the WPT's key focus. For a laser-based system, efficiency is the combination of solar radiation to laser conversion, antenna efficiency, laser transmission, reception antenna, and laser-to-electricity conversion efficiency [126].

#### 5.1.2 Microwave

In this technology, RF frequencies of 300 MHz to 300 GHz are used to transport energy. Since Maxwell's invention of microwave power transmission, it has been used in several applications. The optimal choice of power beaming frequency is a factor in the trade-off between the satellite orbit, satellite size, power beaming efficiency and level transmitter, the transmitter and receiver diameter, and the thermal limits on the panel in the center of the received beam [118]. Due to the atmospheric window for minimizing transmission losses, the maximum practicable frequency range is between 1 GHz and 10 GHz. Since these frequencies are more likely to be chosen for SBSP, the majority of SBSP investigations have focused on 2.45 GHz and 5.8 GHz. Household appliances also utilize 2.45 GHz, and ETC (electronic toll collection system) utilizes 5.8 GHz [124]. In Table 5 we discuss the basic difference between these parameters.

**Table 5:** SBSP Proposed Microwave Transmission parameters [122], [123].

Model	JAXA2 a Model	NASA/DOE Model
Frequency	5.8 GHz	2.45 GHz
Transmitting Antenna Diameter	1.93 km	1 km
Magnitude Reduction	10 dB Gaussian	10 dB Gaussian
Power Output	1.3 GW	6.72 GW
Highest Power Density in the center	114 mW/cm2	2.2 W/cm2
Least Power Density Near the Edge	11.4 mW/cm2	0.22 W/cm2
Power/antenna (Number of elements)	Max. 1.7 W(1,950 million)	Max. 185 W(97 million)
Peak Power Density	100 mW/cm2	23 mW/cm2
Efficiency of Collection	87%	89%

A microwave transmitter sends electromagnetic waves to a remote receiving antenna. A Rectenna combines a rectifier with a receiving aerial. Due to high frequencies, antennas and circuits must take wavelengths into account [118], [126]. The microwave energy transmission mechanism is depicted in Fig. 13b. The transmission efficiency of an MWPT system is determined by the microwave conversion, transmitting antenna, receiving antenna, conversion circuit, and rectifier efficiencies [126]. Other considerations include the efficiency and power capacity of the power electronics utilized in WPT and reception. This is determined by the semiconducting base material, design, improved array impedance matching, and operating frequency [90]. Phase control can also be used to steer phased-array beams. Retrodirective systems can receive a single piloting signal from the site that is receiving power and then transmit power by precisely focusing the beam in the general region where the pilot signal originated from.

Despite what the block diagram may suggest, laser and microwave systems do not share essential characteristics. WPT system approaches are compared in Table 6. WPT research has increased recently. A thorough analysis of current studies indicates how these initiatives are fundamentally different from one another, as well as the various strategies used to address some related problems. WPT's challenges are also shown in these investigations. Table 7 summarizes wireless power transmission's major attempts.

**Table 6:** Difference Between Microwave Power Transmission & LASER Power Transmission [82], [124], [125].

Microwave	Laser
It is simple to deploy incremental advances that conflict with radio transmissions.	No incremental developments.
The ability of transmission is independent of climate.	Disperses in cloudy conditions Consequently, the climate has a noteworthy influence on its transmission capacity.
Since the wavelength is long, cm, or mm, big and heavy components are required.	Due to the short wavelength, transmitter and receiver components must be compact.
Hard to transmit from outer space.	Comparatively simpler to carry.
May or may not require protection based on the intensity of the beam.	Required a precaution to preserve health, including eye protection.
Perform at 5.8 GHz or 2.45 GHz (reduced absorption by the atmosphere)	The wavelength is 1030 nanometers (Er: YAG laser)
The size of components makes scaling down difficult.	Able to ramp up/down (narrow beamwidths possible)
Large rectenna with klystron energy converters constitute the receiver.	Numerous small lasers aimed at the very same object may be required to maintain acceptable energy levels, rather than a single huge laser.
Massive, therefore the system must be large.	Small, permits adaptable systems integration.
Interference with radio frequency	No interference except some related to astronomy.
Permeates clouds and light precipitation.	Prevented by clouds (need decent area).
Legal issues with FCC, NTIA, and ITU.	Given high energy density legal related to ABM treaty.
Rectenna is useful for only SBSP and long-distance transmission.	PV array for both WPT and antenna is only applicable for SSP solar power.

**Table 7:** Study on notable contribution of several papers in recent years.

Title	Inference
Solar Based Wireless Power Transfer System [127]	Solar-powered WPT systems are evaluated in this paper. To maximize energy transmission, coil configurations had been modeled. The optimal coil design for solar-powered receiver and transmitter coils has been evaluated.

A constant efficiency of rectifying circuit in an extremely wide load range [128]	This research optimizes the microwave power efficiency at 2.45 GHz through rectenna conversion implementing maximum power point tracking (MPPT).

Development of Wireless Energy Transfer Module for Solar Energy Harvesting [129]	This paper shows that WPT modules with flat spiral coil transmitters and receivers are more efficient than circular loop designs. Despite its lower Q factor, flat spiral coils are more efficient and require fewer construction parameters. A higher Q factor improves spiral coil efficiency. The wireless transmission module can receive up to 80% of PV cell energy.

A space-to-space microwave wireless power transmission experiential mission using small satellites [130]	This paper suggests employing smaller satellites to test and demonstrate MWPT with the space solar microwave power transfer system. The project will demonstrate WPT efficiency and practicality for powering spacecraft beyond Earth's orbit.

Transmission Line Resistance Compression Networks and Applications to Wireless Power Transfer [131]	This research presents multiway transmission line resistance compression networks (TLRCNs) for radio frequency-to-dc conversion and WPT. The peak efficiency of a 4-way TLRCN network at 2.4 GHz RF frequency was 70%.

Microwave and Millimeter Wave Power Beaming [123]	This study explores the fundamentals of power beaming. This document describes the first in-orbit test and the construction of multiple in-orbit projects to illustrate SBSP's essential elements and the health effects of a 100-kW mm transmitter. The paper's historical summary reveals increased activity in recent years. This study also examines rectenna design evolution.

Safety of Wireless Power Transfer [122]	WPT technology and applications' safety effects will be examined in this study. It shows radio frequency energy harvesting over long distances' intriguing research potential.

A long-distance high-power microwave wireless power transmission system based on asymmetrical resonant magnetron and cyclotron-wave rectifier [132]	This paper analyzes a far-distance MWPT system for high load using a cyclotron-wave rectifier and asymmetrical resonant magnetron. The analysis-based system architecture is presented in this study.

History and Innovation of Wireless Power Transfer via Microwaves [133]	This paper explains MWPT's evolution. This paper also discusses fundamental, applied, and regulatory technologies. These topics are divided into broad and narrow beam MWPT.

Satellite solar wireless power transfer for baseload ground supply: clean energy for the future [90]	This study investigates satellite solar power station (SSPS) base-load electricity generation. It compares 2.45 GHz and 5.8 GHz frequencies and transmitter antenna size estimations for a 10 GW model.

Investigation of wireless power transfer applications with a focus on renewable energy [124]	This study provides evidence for the enhancement of renewable energy utilization via WPT innovation, application, and development challenges along with how laser-based and microwave systems have become viable WPT solutions and their key differences.

### 5.2 Recent developments in wireless power transmission technologies

Although there has been a lot of work related to wireless power transmission technology since the concept was established by Nikola Tesla, in recent years the work has intensified. There has been a lot of practical demonstration by many countries in recent years. Table 8 goes over the recent successful demonstration of wireless power transmission.

**Table 8:** Country-wise piratical demonstration of wireless power transmission in recent time.

Country	Activities
JAPAN	Mitsubishi Heavy Industries, Ltd. (MHI) in Japan achieved an MWPT record by sending 10 kW of 2.45-GHz RF power 500 meters utilizing Kyoto University-designed phase-controlled magnetrons with arrays of 8 m x 8 m for the transmitter [134], [135]. Ministry of Economy, Trade and Industry (METI) SPS team conducted a horizontal aerial microwave beaming test on March 8, 2015. Four phased array panels equipped with 2.45 GHz retro-directivity transmit 1.8 kW of 5.8-GHz microwave radiation 55 m to the rectenna array and obtained approximately 335 W DC [136]. In May 2019, METI successfully powered a drone wirelessly utilizing the prior phased array and built a lightweight rectenna with 17 circular microstrip antennas. It received 4 kW/m2 of microwave radiation with 60 W DC for 10 m and 42 W DC for 30 m. METI SPS started building the sandwich model with a microwave-phased array and PVs in 2019, with completion planned in 2024 [137]. Tsukuba University, Japan created a 303-GHz antenna in 2018 utilizing a GaAs Schottky diode [138].

SOUTH KOREA	In 2019, American and Korean researchers transmitted electricity at 10 GHz to an airship, enabling it to navigate at 7 mph inside the High Intensity- Radiated Fields chamber at NASA Langley Research Center [139]. A Korean company has developed X-band microwave power transmitters with 2304 GaN MMIC elements and 1.5 kW of transmit power [123]

CHINA	China plans to build the first functional SPS facility with an experiment base built on December 6, 2018, that will cover 130,000 m^2^ and cost $30M. In 2020, Chongqing University announced the phase-controlled source, transmit antenna, and power subsystem in relation to this [123]. In 2018, Xi'an researchers described concentrated CP microwave power beaming with a conversion efficiency of 66.5% RF-to-DC. In December, the ZhuRi (Chase the Sun) SPS project debuted a ground-based OMEGA SPS system. [123]. In 2020, 1 kW of electricity was beamed at 35 GHz over 300 m utilizing a spatiotemporal antenna, mm-Wave tube transmitter, and GaN diode rectenna [123].

UNITED STATES	Caltech along with Northrop Grumman created a high-efficiency, lightweight PV-array system in 2018 [140]. Northrop Grumman is collaborating with the Air Force Research Laboratory to develop an SSP system on the Space Solar Power Incremental Demonstrations and Research project. In 2020, Caltech revealed many advances in SPS power beaming, including adaptable RFIC-based phased arrays with dynamic calibration and timing devices for large-scale phased array synchronization [141]. The US Naval Research Laboratory (NRL) launched PRAM-FX on an Air Force X-37B on May 17, 2020. 12-inch square PRAM-FX tiles convert solar energy to RF microwave power [123].

### 5.3 Applications of wireless power transmission in other fields

The impact of WPT extends well beyond SBSP. MWPT can wirelessly transform the grid [142]. By converting to a wireless grid, we can eliminate e-waste and deliver electricity 24 hours a day, seven days a week. This may benefit IoT devices. As IoT devices continue to shrink, WPT may assist minimize system size and enable their deployment in locations where cable transmission is impractical [143]. In the future, a transmitter inside the home will transfer power to all household appliances and receive it via receiving devices in each appliance [142], [144]. EVs may be charged wirelessly [142], [144]. To save fuel and reduce pollution, buses, trains, airplanes, UAVs, and AUVs can be powered wirelessly [144], [145]. WPT may have a significant impact on the medical industry. Most medical implants do not require charging after they are implanted. In some medical applications, a WPT transmitter may be implanted externally while the receiver is placed within. Hence, battery replacement is avoided [142].

### 5.4 Wireless power transmission challenges

#### 5.4.1 Biological effect

There has been great concern over the effects of radiation on human life. Particularly, there have been debates over the effects of 50/60 Hz and frequencies greater than one gigahertz. Because some countries use 50, others use 60 Hz for transmission line but the microwave frequency wave will be withered 2.4 or 5.8 GHz. Since the majority of research on the biological impacts of microwaves focuses on relatively minimal exposure and the low magnetic flux densities typical of residence situations, no appreciable effects will be seen on cells or animals. For, higher frequency substantial studies are not present for any claim to be made with 100% certainty.

For humans, objects, and animals subjected to the beam, the power concentration exhibited may be dangerous. Various organizations, such as the IEEE [146], International Commission on Non-Ionizing Radiation Protection (ICNIRP) [147], have defined safety criteria for restricting continuous human contact with particular power density limits for both laser energy beaming and microwave power beaming. According to the details in these documents, limits apply to both power density and absorbed energy. The SAR (Special absorption rate) threshold determines the majority of adverse effects on humans. The ICNIRP exposure guidelines for GHz beam are 50 W/m^2^ for occupational exposure and 10 W/m^2^ for the general population [148], [149]. Because most of the experiments take place in laboratory-controlled environment it is hard to determine the effect these will have when this technology will be used on a commercial level.

#### 5.4.2 Integration with existent structure

Microwaves beam at 2.45 GHz and 5.8 GHz, which is the spectrum allotted to radio services under the ITU-R Radio Standards. It also falls inside the allocated ISM frequency channels. Similarly, MPT has no assigned frequency band, hence it utilizes the ISM band [148], [149].

#### 5.4.3 Interference with atmosphere

Considering the absorption and scatter effect of air and rain as well as the irregular ratio of air refraction, previous studies indicate that microwaves have a negligible impact on the atmosphere. The microwave spectrum absorption of oxygen is about 0.007 dB/km which is very limited [148], [149].

#### 5.4.4 Need for improved standards & new bandwidth allocations

New standards need to be introduced to improve rules and regulations for communication and electromagnetic interference issues affecting telecommunications, connectivity, and electrical power systems [148]. Improved bandwidth allocations are needed to provide for improved power transfer rates and the maximum feasible separation between power, communications, and navigation satellites [148], [145].

### 5.5 Steps to overcome limitations of wireless power transmission

To minimize the amount of electromagnetic interference and radio frequency interference between various types of research satellites and SBSP systems, the current techniques must be enhanced. SBSP transmissions from space to rectennas on Earth must adhere to several health and safety regulations [148], [122]. International Telecommunication Union (ITU) is responsible for distributing new licensed spectrum to ground stations for space-to-Earth power transmission. New standards for SBSP rectennas that address the issue of possible reflected radiation back out to space, as well as a more thorough update of satellite navigation, and user interface design to reduce interference need to be addressed. National legislation must be compliant with space treaties in order to establish new criteria for the active de-orbiting of space debris; the growing size of SBSP systems exacerbates the dangers connected with space debris to SBSP systems [148], [145].

## 6 Spillover benefits: applications & opportunities

SBSP's ability to transport vast quantities of baseload electricity straight to remote locations and its support of space manufacturing, which cuts CO2 emissions, make it an attractive choice [150]. Its use is widespread, and these technological advances can assist other areas. Some of the main applications are:

### 6.1 Diversifying electric grid & remote power

SBSP platforms can enhance energy security without a unified grid and provide flexible and reliable baseload power moving Earth towards cleaner energy independence [90]. By continuously accessing one-third of Earth's surface, an SBSP platform in geostationary orbit may send energy to distant places. An SBSP system can immediately reroute power transmission during a nationwide blackout. In non-crisis settings, the SBSP platform can mitigate load surges across time zones because energy consumption peaks in the morning and evening [23], [25]. SBSP can supply energy to offshore desalination facilities, offshore carbon capture plants, and other off-grid places [151]. Solar energy use in electricity is rising, while space holds 10 – 40 times more energy per square meter [152]. Hence, an SBSP platform can increase grid resilience and encourage renewable energy while constructing new transmission infrastructures.

### 6.2 Deep space exploration & space manufacturing

Solar energy derived from space is a viable alternative energy source for space operations. It can be diverted to multiple locations and scaled up or down to fulfill altering mission objectives [153]. This may also benefit the space manufacturing industry by providing them with a sustainable and long-lasting energy source [154].

### 6.3 An alternative energy source

The SBSP offers immediate standby energy production to places where an alternative to the main energy system is necessary, but solar, wind and other inherently variable resources require backup electricity production. It means keeping oil and coal power plants running at total capacity to provide energy. Due to unpredictable weather, the backup system and operators must constantly be ready [105].

### 6.4 Disaster response

SBSP may provide energy to regions whose ability to generate electricity is hindered by natural disasters. After a natural disaster, it takes time to repair, reconnect, and restore it to its previous condition. Hence, SBSP might serve as a replacement for the damaged power plant, speeding the delivery of electricity to hospitals, families, and businesses, thereby saving lives and properties [155].

### 6.5 Benefits in other sectors

Several industries, like consumer electronics and electric car charging, are projected to benefit from the potential to transmit useful electricity over long distances without wires. This could reduce the need for long-distance lines, hence minimizing electronic waste. Developing high-powered microwave devices would increase the efficiency of electric switching in power networks and radar systems. For various electrical power applications, high-efficiency power electronics will allow for low-cost, high-volume production. HCPV (Heliostat Concentrator Photovoltaic) and comparable semiconductor technologies will benefit space and terrestrial solar cell technologies. The entry of private firms into the space race hastens the commercialization of space. This promotes the provision of commercial services by space commerce firms. [156]. This exciting project may inspire a new generation of engineers covering a wide range of STEM topics.

## 7 Challenges, risks and concerns

Space-based solar power development is complex due to the scale and integration requirements of the system. When completed, the solar power satellite would be the largest and heaviest in orbit. The performance of the power-to-mass ratio (kW/kg) is critical. Low system mass and proof of power transmission from solar photovoltaic panels in space to the electrical grid are required.

### 7.1 Technological

SBSP can be developed without any scientific or technological breakthroughs, but significant advances are needed in many fields. There is no getting around the fact that this is a big roadblock to actualizing the dream. Due to the size and mass of the system, crucial components must be constructed in orbit, yet humans have never reached upper Earth orbit. A structure of this magnitude will require assembly and robot maintenance in orbit. Current rocket and thruster technology makes it difficult to transfer the structure from a lower orbit to a higher orbit. Smaller, lighter panels make construction simpler and less expensive. To recoup the whole cost and generate a profit, the satellite's operational lifetime must be considered. This suggestion was motivated by the low efficiency of conventional solar plants; hence, wireless power transfer technology and efficiency must be enhanced. For wireless power transfer, efficiency is crucial, as the lower efficiency of conventional solar plants was the impetus behind the development of this project. Cybersecurity is challenging. Current software issues may compromise precision and performance. Software bugs have become increasingly prevalent in recent years, and they are a potential source of both decreased precision and altered strength. To prevent satellites from transmitting at an unsafe power level, they must be built with redundancy in mind [20], [23].

### 7.2 Economic

Existing or anticipated economic elements and capabilities impact the economic viability of SBSP. In the long term, the project's cost will be the greatest obstacle to its financing. Theoretically, potential space solar energy has been commercially impractical for decades. Two technological factors are driving space-based solar power-enabling improvements: substantially decreased space launch costs and cheaper prices for space components as a result of innovations in manufacturing. However, the overall cost remains high, and effort is required to bring it down. The levelized cost of energy (LCOE) compared to other renewable technologies should be extensively researched. The project's industrial features can have a significant impact on its economic feasibility [20].

### 7.3 Political

The project will be driven by both local and global politics. Locally, integration with existing infrastructure and energy policies will be tough. As the project will take decades to finish and yield results, the time of development should be carefully planned. Earth-receiving stations, especially the rectennas, will require a large amount of land, which must be meticulously planned. The project necessitates space and land security. By sharing expenses and benefits, international collaboration can ensure the success of a project and stimulate the economy [21].

### 7.4 Environmental and safety

Although the wavelength is harmless, long-term exposure to a rectenna may harm the environment. Together with project durability and safety, viability should be evaluated. Safety concerns preclude the use of lasers. The structure's decommissioning may generate space debris. Also, the whole system must be protected from space debris, which might potentially cause structural damage [21], [157].

### 7.5 Social

Large-scale projects require public support. Safety and security concerns exist with wireless power transfer. There are several safety and security concerns about wireless power transfer. Like, how will wireless transmissions affect individuals? Will aircraft, birds, or land structures be endangered by this? These concerns can be put to rest with the knowledge that SBSP systems are designed to be completely risk-free, posing no threat to any living or nonliving entities, including humans, birds, airplanes, structures, or anything else. Truth be told, these shows aren't that different from the ones we see every day. Transferring energy from a solar power satellite often makes use of low-powered microwave transmission. The wavelengths are identical to those of kitchen appliances, but the signal strength is substantially weaker. The Federal Communications Commission (FCC), which regulates wireless transmissions in the United States, warns that microwave ovens “should not be confused with the lower-energy, non-ionizing radiation” from low-power transmissions. A public demonstration that addresses safety issues and raises awareness might be beneficial [21], [154].

### 7.6 Legal

The majority of nations have vague SBSP regulations. Lack of transparency may discourage inventors and businesses, putting them in danger. Wireless power transmission is a crucial regulatory issue that would come within the purview of government agencies and need worldwide coordination via the International Telecommunications Union (ITU). The ITU is an international diplomatic organization in which states collaborate to determine which applications are favored for a specific frequency range. The ITU frequency chart acts as an agreement for transmissions that traverse international boundaries. Wireless communication is mostly regulated for device and broadcast interference. Countries must recognize that wireless power transmission fits into the standard structure. To prevent interference and identify affected equipment and communications, it is necessary to negotiate and test frequencies. The orbital allotment of the satellite adds to the legal complication. Space is not owned; however, the size of the proposed structure may raise difficulties [158].

## 8 Space-based solar power alternatives

SBSP faces considerable challenges, including the prohibitive costs associated with deploying and maintaining solar arrays and transmitters in outer space. However, numerous renewable energy technologies demonstrate promise in efficiently meeting the demands for sustainable energy. In recent years, solar PV capacity has increased significantly, aided by decreasing costs. Although ground-based solar PV is less efficient than space-based collection, it circumvents the enormous initial costs associated with launching and deploying space infrastructure. Even in densely populated nations, there is ample undeveloped land, and solar panel technology is advancing consistently; therefore, ground-based solar PV has ample room to continue expanding sustainably. Alternative wind fields may be located offshore, where winds are more consistent and powerful than those that are generated on land. Presently, floating foundations for wind turbines allow for the exploration of the vast offshore wind potential, even in the depths of the ocean [159].

However, nuclear power offers a reliable and carbon-neutral source of base-load electricity to offset the variability of renewable energy sources. Additionally, technological advances in nuclear power, such as tiny modular reactors, may render the industry more adaptable and cost-effective. Concerns regarding accidents and the disposal of radioactive waste persist; nevertheless, nuclear power presently provides approximately 10% of the global electricity demand. Despite encountering challenges related to financial investment and public opinion, nuclear power remains a reliable source of low-carbon electricity that is anticipated to make a substantial contribution towards fulfilling the escalating energy requirements of the future [160]. An optimal approach could involve the integration of various renewable technologies, such as solar PV, offshore wind, and geothermal, alongside continuously increasing storage options, as opposed to relying on a single solution. A balanced portfolio permits concessions on the following fronts: capacity, environmental impact, cost, and efficiency [161].

## 9 Conclusion

SBSP could be an essential factor in achieving net zero by 2050 with our current renewable technology, which seems like a distant dream. The overall system concepts and other factors like cost and technology for making SBSP possible have come a long way. There are still several questions about the overall concept. The power ratio of space solar power to traditional solar power is 40:1. Traditional solar power does not provide power 24/7 and depends on weather conditions, whereas SBSP provides baseload power 24/7, independent of weather conditions. There is also the case of using the rectenna area, which needs to have a contiguous area of the order of km in diameter. Traditional solar panels also take up a large amount of space with a low power-to-area ratio. These solar panels also have a lot less life span than the ones used in the SBSP.

The expenses associated with space exploration have significantly decreased in recent years due to technological developments like as the development of reusable rockets and reduced manufacturing costs facilitated by improved automation. This significantly enhances the economic viability of space-based solar power compared to its previous feasibility. Furthermore, alongside the decrease in launch expenses, we have witnessed significant advancements in the solar cell technology itself. Advancements in power electronics are enabling the development of solar cells that are thinner, more efficient in converting sunlight into electricity, and with extended lifespans. Contemporary solar cell designs effectively resolve common problems that formerly impacted solar cells in space applications, such as excessive heat accumulation and challenges in dissipating heat. Given the decreased expenses associated with space travel and the progress made in solar cell technology, space-based solar power has now become a viable option for meeting a significant amount of the global energy needs, if it is deployed on a large scale. The convergence of reduced expenses and enhanced technology has transformed previously deemed unfeasible concepts into auspicious systems that have the potential to provide environmentally friendly and sustainable energy to people worldwide. What was formerly seen as an excessively costly or technically unachievable energy solution now needs careful evaluation and advancement. However, harnessing the promise of SBSP necessitates tackling substantial technical obstacles in wireless power transmission.

The potential of space-based solar power is considerable; however, in order to fully harness its advantages, sustained and persistent long-term efforts would be required. Further research and development are needed in the following areas: optimizing the performance and reducing the mass of solar cells to decrease launch costs; advancing wireless power transmission technologies to ensure dependable energy transmission over long distances; refining photon-to-electron conversion systems; and developing remotely maintainable structures that are both lightweight and sturdy. Although progress has been made in these areas, additional endeavors are necessary. Conducting trials on scaled-down SBSP systems, such as employing them to supply power to missions in closer proximity to Earth, can aid in validating model accuracy and assessing the feasibility of the technology prior to the establishment of a full-scale power plant. Additional investigation is necessary in order to improve cost effectiveness, scalability, and reliability. The potential benefits of participating in global collaboration to establish policy frameworks and standards include the stimulation of public and private sector investment. The optimistic projection for an ample supply of pure baseload electricity derived from space by 2050 is substantiated by comprehensive investigations in critical domains and governmental endeavors focused on technological and infrastructure progress.

Throughout the paper, we discuss a wide range of topics on SBSP. An in-depth discussion is given on various issues, including its early history and the reasons why, given the qualities of SBSP, it is now more possible than it ever was before. One of the main points of this paper is discussing the whole system view, where we have a detailed idea of a generalized system with other factors. We also discuss some prominent SBSP designs. The applications and benefits for other sectors with challenges and risks are discussed in detail. These ranges are not only technical but also legal and social. In conclusion, it is possible to assert that the SBSP can resolve the impending crisis in the energy sector if given the appropriate amount of attention to detail in terms of planning and direction.

## CRediT authorship contribution statement

**Khandoker Shahjahan Alam:** Writing – original draft, Resources, Investigation, Conceptualization. **A.M.A. Daiyan Kaif:** Writing – original draft, Resources, Investigation, Conceptualization. **Sajal K. Das:** Supervision, Project administration. **Sarafat H. Abhi:** Validation. **S.M. Muyeen:** Supervision. **Md. Firoj Ali:** Validation. **Zinat Tasneem:** Visualization. **Md. Manirul Islam:** Formal analysis. **Md. Robiul Islam:** Formal analysis. **Md. Faisal R. Badal:** Writing – review & editing. **Md. Hafiz Ahamed:** Writing – review & editing. **Subrata K. Sarker:** Writing – review & editing. **Prangon Das:** Writing – review & editing. **Md. Mehedi Hasan:** Writing – review & editing.

## Declaration of Competing Interest

The authors declare that they have no known competing financial interests or personal relationships that could have appeared to influence the work reported in this paper.

## Data Availability

The authors do not have permission to share data.
